# Reagentless Glucose Biosensor Based on Combination of Platinum Nanostructures and Polypyrrole Layer

**DOI:** 10.3390/bios14030134

**Published:** 2024-03-04

**Authors:** Natalija German, Anton Popov, Almira Ramanaviciene

**Affiliations:** 1 Department of Immunology and Bioelectrochemistry, State Research Institute Centre for Innovative Medicine, Santariskiu 5, LT-08406 Vilnius, Lithuania; anton.popov@chgf.vu.lt; 2 NanoTechnas—Center of Nanotechnology and Materials Science, Institute of Chemistry, Faculty of Chemistry and Geosciences, Vilnius University, Naugarduko str. 243, LT-03225 Vilnius, Lithuania

**Keywords:** glucose, glucose oxidase, 1,10-phenathroline-5,6-dione, platinum nanostructures, polypyrrole

## Abstract

Two types of low-cost reagentless electrochemical glucose biosensors based on graphite rod (GR) electrodes were developed. The electrodes modified with electrochemically synthesized platinum nanostructures (PtNS), 1,10-phenanthroline-5,6-dione (PD), glucose oxidase (GOx) without and with a polypyrrole (Ppy) layer—(i) GR/PtNS/PD/GOx and (ii) GR/PtNS/PD/GOx/Ppy, respectively, were prepared and tested. Glucose biosensors based on GR/PtNS/PD/GOx and GR/PtNS/PD/GOx/Ppy electrodes were characterized by the sensitivity of 10.1 and 5.31 μA/(mM cm^2^), linear range (LR) up to 16.5 and 39.0 mM, limit of detection (LOD) of 0.198 and 0.561 mM, good reproducibility, and storage stability. The developed glucose biosensors based on GR/PtNS/PD/GOx/Ppy electrodes showed exceptional resistance to interfering compounds and proved to be highly efficient for the determination of glucose levels in blood serum.

## 1. Introduction

Biosensors have a broad spectrum of applications covering food and pharmaceutical industries, environmental monitoring, and clinical diagnostics [1,2,3,4,5]. The first electrochemical biosensor based on the Clark oxygen electrode with immobilized glucose oxidase (GOx) was developed by Clark and Lyons in 1962 and was adapted for glucose physiological monitoring in blood plasma [6]. Glucose, an aldose monosaccharide, is considered one of the most important molecules for most organisms. It plays a key role in photosynthesis, respiration, and various metabolic processes [1]. Diabetes mellitus (often known as diabetes) poses a significant global public health challenge [2]. High blood glucose concentration damages the retina, kidneys, nerves, and circulatory system and is the leading cause of blindness among US adults between the ages of 20 and 74 [2]. Diabetes is responsible for an estimated 3.5% of deaths from cardiovascular disease, cancer, and chronic respiratory diseases [4]. Enzymatic electrochemical biosensors based on biospecific enzyme reactions are the most widely employed biosensors for glucose monitoring due to their cost-effectiveness, stability, rapid response, and ease of use [1,2,3,4,5]. Glucose oxidase, which consists of two identical protein subunits containing a single flavin dinucleotide (FAD) coenzyme molecule in each subunit, is considered one of the most popular biocomponents for the development of glucose biosensors due to its specific activity, stability, and high selectivity [6,7].

Direct electron transfer between the FAD of glucose oxidase and the surface of the electrode is kinetically challenging due to the deeply embedded cofactor in the GOx redox center [2]. One common approach to overcome this kinetic difficulty is to use redox mediators to efficiently transfer electrons from the GOx redox center to the electrode [7]. Heterocyclic quinoid compounds, such as 1,10-phenanthroline-5,6-dione (PD), can act as proton and electron acceptors towards the regeneration of FAD. They are characterized by fast electron transfer kinetic, high stability in their oxidized and reduced forms, and are suitable for the development of reagentless biosensors and biofuel cells [8,9]. In electrochemical sensors, a molecule of quinone is first reduced to a molecule of deprotonated hydroquinone and then re-oxidized at the working electrode [9].

The conducting polymer, polypyrrole (Ppy), offers advantages for practical biosensor applications, including suitability to be a matrix for enzyme immobilization and increased operational stability [10,11,12]. Enzymatic polymerization is a suitable method to form a Ppy layer on the electrode surface [11]. Hydrogen peroxide (H_2_O_2_), which is a product of a GOx enzymatic reaction, initiates the synthesis of Ppy [2,11]. A uniform layer of polymer covers the enzyme and protects the electrode from electrochemically active materials present in various samples [10]. The electrical conductivity, degree of resistance to interfering and electroactive species, and storage stability of biosensors based on polymer nanocomposites can be improved by embedding noble metal nanomaterials into the polymer matrix during electrochemical [13,14,15] or enzyme-mediated [16] polymerization. Nanomaterials provide a suitable microenvironment for GOx immobilization, retaining its biological activity [17,18], facilitate the heterogeneous reaction [7] and enhance the electron transfer rate [19,20,21] with their large electroactive surface area [21,22,23,24].

Palladium [15], platinum or gold nanoparticles (PtNPs [24,25,26,27,28,29,30,31] or GNPs [16,17,32]), platinum nanoclusters (PtNCs) [33], platinum nanostructures (PtNS [34,35,36]) or gold nanostructures [19,37,38,39,40,41,42,43], and carbon nanotubes [9,44] are used in various fields of modern science, medicine, and industry [4,5]. Nanoparticles have applications in radiation and photothermal therapy, nanodiagnostic [25], and genosensor [20]. They are used for ascorbic acid [9], hydrogen peroxide [27,44], methanol [28] and glucose detection [16,19,43], as well as for enzyme immobilization, in electrocatalysis, and in the development of light-emitting structures and solar and fuel cells [26,36,41].

In recent years, the fabrication of PtNS and their application in industrial and clinical assays have received much attention due to their high conductivity, stability, and oxidation resistance [36]. The fabrication of 2D or 3D platinum nanomaterials can be carried out with the reduction of PtCl_6_^2−^ ions to Pt atoms in a hydrogen flow at 300 °C [34], electrochemical deposition [14,22,28], and photocatalytic seeding [35]. 2D arrays of Pt nanocrystals can be assembled using the Langmuir-Blodgett method [29]. The morphology of PtNS depends on the duration of synthesis, the concentration of H_2_PtCl_6_, the supporting electrolyte and solvent used for PtNS preparation [34,35,36]. The galvanic replacement reaction at −0.60 V vs. Ag/AgCl_(sat.KCl)_ was applied for the formation of 3D nanoporous platinum structures with and face-centered cubic crystal structure [22]. Platinum aggregates (size 2–5 nm) were deposited on the surface of polycrystalline boron-doped diamond with cyclic voltammetry (CV) from +0.60 to −0.25 V at a scan rate of 0.1 V/s or with constant potential amperometry (CPA) at −0.2 V deposition potential vs. Hg/Hg_2_Cl_2(crystal KCl)_ for 5 s [28] and platinum agglomerates (3–10 nm)—on nanoporous gold electrode at −0.2 V vs. Ag/AgCl_(0.1M KCl)_ from 1 to 60 s [45]. Clump-like crystal aggregations of PtNS were characterized by the highest electrocatalytic performance and activity compared to leaf-like flake and spherical clusters, which allows their use in fuel cell and biosensor designs [36]. Highly sensitive glucose biosensors were developed using one-step laser-scribed PtNPs nanostructured 3D porous laser-scribed graphene (LSG) with a thin film of hydroxyethyl cellulose (HEC) and immobilized GOx (LSG/HEC-PtNPs/GOx, 69.64 μA/(mM cm^2^)) [24] and an indium tin oxide (ITO) electrode modified with on a poly(2-vinylpyridine) (P2VP) grafted metal organic framework (UiO-66-*g*) with ultrafine PtNCs nanocomposite (ITO/PtNCs_(1.76nm)_/UiO-66-*g*-P2VP, 199.58 and 74.45 μA/(mM cm^2^) for 0.01–5 and 5–18 mM of linear range (LR), respectively) [33].

Despite the advances in the development of glucose biosensors, the search for improvements and innovative solutions in this field continues [3,4,5]. The main idea of this work was the combined application of PtNS and Ppy to improve the analytical performance of glucose biosensors. The effect of Ppy on the current responses toward glucose was evaluated. The performance of electrochemical biosensors based on GR/PtNS/PD/GOx and GR/PtNS/PD/GOx/Ppy electrodes with respect to analytical characteristics, stability, and potential applications for glucose monitoring in blood serum are presented and discussed.

## 2. Materials and Methods

### 2.1. Materials

D-(+)-glucose, D(+)-saccharose, D(+)-xylose, D(+)-galactose, D(+)-mannose, D(−)-fructose, hexachloroplatinic (IV) acid hexahydrate (H_2_PtCl_6_·6H_2_O), and glucose oxidase (type VII, from *Aspergillus niger*, 208 units/mg protein) were acquired from Carl Roth GmbH+Co.KG (Karlsruhe, Germany) and Fluka (Buchs, Switzerland), respectively. Graphite rod (diameter of 3 mm), hydrochloric acid, and 1,10-phenathroline-5,6-dione were obtained from Sigma-Aldrich (Saint Louis, MO, USA), L-ascorbic acid (AA) and uric acid (UA)—from AppliChem GmbH (Darmstadt, Germany), potassium nitrate (KNO_3_) and pyrrole—from Acros Organics (Morris Plains, NJ, USA). Alfa alumina powder (Al_2_O_3_, diameter of 0.3 μm, type N) was purchased from Electron Microscopy Sciences (Hatfield, MA, USA), 25% glutaraldehyde (GA) solution—from Fluka Chemie GmbH (Buchs, Switzerland), potassium chloride (KCl)—from Lachema (Neratovice, Czech Republic), potassium hydroxide, sulfuric acid (H_2_SO_4_), sodium acetate trihydrate (CH_3_COONa·3H_2_O)—from Reanal (Budapest, Hungary), hexaammineruthenium (III) chloride (Ru(NH_3_)_6_Cl_3_)—from Fisher Scientific (Waltham, MA, USA). The solution of sodium acetate (SA, pH 6.0) buffer was prepared by the mixing of 0.05 M CH_3_COONa·3H_2_O and 0.1 M KCl; the solution of PD—in 96% of ethanol from Gintarinė vaistinė (Vilnius, Lithuania). The pyrrole was filtered through a column (5 cm long) with Al_2_O_3_ before use.

### 2.2. The Methodology of GR Electrodes Modification

**Figure 1:**
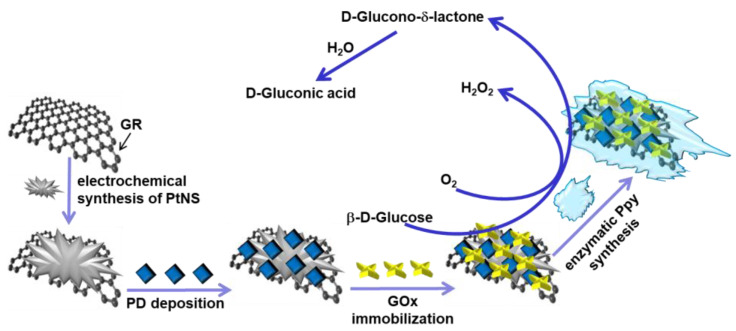
Schematic illustration of the fabrication and operation of glucose biosensors based on GR/PtNS/PD/GOx and GR/PtNS/PD/GOx/Ppy electrodes.

Two types of electrodes, (i) GR/PtNS/PD/GOx and (ii) GR/PtNS/PD/GOx/Ppy, were fabricated as shown in Figure 1. Before modification, GR (0.071 cm^2^ area) was carefully polished on fine emery paper and then polished with a slurry of alfa alumina powder. After washing with distilled water and drying at room temperature (20 ± 2 °C), polished GR was sealed into a silicone tube.

To fabricate GR/PtNS/PD/GOx electrodes, the polished surface of GR was coated with electrochemically synthesized PtNS from a solution of 6 mM H_2_PtCl_6_·with 0.1 M KNO_3_ using the CPA method for 100 s at an applied potential of −0.2 V vs. Ag/AgCl_(3M KCl)_ (Metrohm, Herisau, Switzerland). Then, 4.5 μL of 38 mM PD solution was added to the electrode covered with GR/PtNS and dried at +20 ± 2 °C. The amount of 3 µL of 25 mg/mL GOx solution was dropped onto the pre-modified electrode and dried at room temperature. The modified electrode was then stored for 15 min in a closed vessel over a 25% GA solution at +20 ± 2 °C to cross-link GOx molecules. To prepare the GR/PtNS/PD/GOx/Ppy electrode, modified with PtNS, PD, and GOx GR electrode was stored for 5 h in the polymerization solution consisting of 0.05 M of SA buffer (pH 6.0), 0.2 M of pyrrole, and 0.05 M of glucose. The enzyme-mediated polymerization was performed at +20 ± 2 °C in the dark. GR/PD/GOx electrode was used to compare current responses toward glucose.

Figure 1 shows the schematic of glucose biosensors, where, in the presence of oxygen, β-D-glucose is oxidized with GOx to D-glucono-δ-lactone followed by hydrolysis in water to D-gluconic acid [7]. Nanomaterials and the redox mediator PD are used to ensure the electron transfer between the redox active site of GOx and the surface of the electrode [8,16]. The polymer layer can increase the stability and improve the anti-interfering ability of the glucose biosensor [11].

### 2.3. The Characterization of Glucose Biosensors Covered by Nanostructures

The electroactive surface area (EASA) of the electrodeposited PtNS was evaluated according to the described methodology [45,46]. To calculate the EASA of PtNS, it is recommended to estimate the Pt-H adsorption peak [46]. Cyclic voltammograms were recorded in 0.5 M H_2_SO_4_ solution in the range from 0.0 to +1.5 V vs. Ag/AgCl_(3M KCl)_ at a scan rate of 0.025 V/s. EASA was calculated using the equation:

(1) $$EASA=\frac{A}{v\;\cdot 210\;µC/{cm}^{2}\;},$$

where *A* is the area of the cathodic peak, *v* is the scan rate (V/s), and 210 µC/cm^2^ is the charge density of hydrogen at the surface of platinum [46].

The morphology and size of PtNS deposited on the surface of the GR electrode were evaluated using a high-resolution Hitachi SU-70 field emission scanning electroscope (FE-SEM, Tokyo, Japan).

The electroactive surface area of GR/PtNS/PD/GOx and GR/PtNS/PD/GOx/Ppy electrodes was evaluated with cyclic voltammetry in the potential range from −0.70 to 0.0 V vs. Ag/AgCl_(3mol L_^−1^_KCl)_ and various scan rates (0.025, 0.050, 0.075, 0.100, 0.125, 0.150, and 0.175 V/s) in the solution of 1 mM Ru(NH_3_)_6_Cl_3_ and 0.1 M KCl. The EASA was calculated according to the following Randles–Sevcik equation [47,48]:

(2) $$I_{p}=2.69\times 10^{5}\cdot n^{\frac{3}{2}}\cdot EASA\;\cdot D^{\frac{1}{2}}\;\cdot C\;\cdot v^{\frac{1}{2}},$$

where *I_p_* is the maximum peak current (A), *n* is the number of electrons transferred in the redox event (Ru(NH_3_)_6_^3+^ + e^−^ ⇆ Ru(NH_3_)_6_^2+^ [47]), D is the diffusion coefficient (9.0 × 10^−6^ cm^2^/s [47]), and *C* is the concentration of electroactive species (0.000001 mol/cm^3^).

### 2.4. The Electrochemical Investigations and the Statistical Evaluation of Glucose Biosensors Performance

Electrochemical measurements were conducted using a three-electrode system: GR/PtNS/PD/GOx or GR/PtNS/PD/GOx/Ppy as the working electrode, 2 cm^2^ platinum spiral BASi Research Products (West Lafayette, IN, USA) as an auxiliary electrode, and Ag/AgCl_(3M KCl)_ as a reference electrode. The GR/PD/GOx electrode was also used in the comparative studies as a working electrode. All electrochemical investigations of glucose biosensors were carried out using a computerized potentiostat/galvanostat Autolab/PGSTAT 302N with GPES 4.9 software (AUT83239). Cyclic voltammograms ranging from −0.60 to +0.60 V vs. Ag/AgCl_(3M KCl)_ at a potential step of 2.5 mV and a scan rate of 0.10 V/s were recorded in an unstirred 0.05 M SA buffer solution (pH 6.0) with 0.1 M KCl. CPA with an applied potential of +0.30 V vs. Ag/AgCl_(3M KCl)_ was used to register current responses of developed electrodes in a stirred (1200 rpm) 0.05 M SA buffer solution (pH 6.0) with 0.1 M KCl.

In order to assess the stability of the developed glucose biosensors, GR/PtNS/PD/GOx and GR/PtNS/PD/GOx/Ppy electrodes were stored over 0.05 M SA buffer solution (pH 6.0) with 0.1 M KCl at +4 °C for up to 42 days.

All CPA measurements were repeated at least three times and presented as the mean value. Software SigmaPlot 12.5 was used to statistically evaluate the intercept, slope, correlation coefficient of the calibration curve, LR, difference of maximal current (Δ*I*_max_), apparent Michaelis constant (*K*_M(app)_), and limit of detection (LOD). The LOD was characterized as the lowest amount of analyte that provided a current response greater than the background current response value plus 3σ. Data analysis was performed via one-way analysis of variance (ANOVA, *p*-value < 0.05).

### 2.5. The Application of Developed Glucose Biosensors for Determination of Glucose in Serum

The impact of various sugars, ascorbic and uric acids on glucose biosensors based on GR/PtNS/PD/GOx/Ppy electrodes was tested in blood serum using the CPA method according to the previously described methodology [49]. For this purpose, the samples of blood serum were diluted 10 times in the solution of SA buffer (pH 6.0) and centrifuged (8 min, 14.6 × 10^3^ xg) using the IEC CL31R Multispeed centrifuge (Aze Bellitourne, Château-Contier, France). The blood serum samples with 10.0 mM glucose before and after the addition of 1.0 mM of saccharose, xylose, galactose, mannose, and fructose were used to evaluate the effect of sugars on the developed glucose biosensor. To investigate the impact of ascorbic and uric acids, measurements were performed in serum with 10.0 mM of glucose without and with 0.01 or 0.1 mM of AA; and with 10 mM of glucose without and with 0.01, 0.05, or 0.1 mM of UA.

## 3. Results

### 3.1. The Characterization of Modified Electrodes

To improve the analytical performance of developed biosensors, the electrode surface was modified with nanostructures [39,49]. The electrodeposited PtNS were employed for this reason in our study. The EASA of PtNS was calculated using Equation (1) (Figure S1), and it was 2.29 ± 0.25 cm^2^. This EASA of the modified electrode was significantly larger compared to the geometric surface area of the GR electrode (0.071 cm^2^).

**Figure 2:**
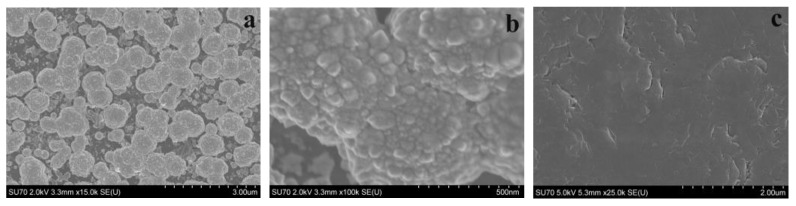
FE-SEM images of electrochemically deposited PtNS on the GR electrode (**a**,**b**) and bare GR (**c**). SEM images were acquired at an accelerating voltage of 2 kV and a magnification of (**a**) 15 k, (**b**) 100 k, or (**c**) 25 k.

The morphology of electrodeposited PtNS on the surface of the GR electrode was evaluated according to the obtained FE-SEM images (Figure 2a,b). The FE-SEM image of bare CR was used as a control (Figure 2c). It can be seen that electrochemically synthesized PtNS were formed as circular, fused aggregates. Small (500–750 nm) PtNS merged into large (3.0–12 μm) aggregates.

**Figure 3:**
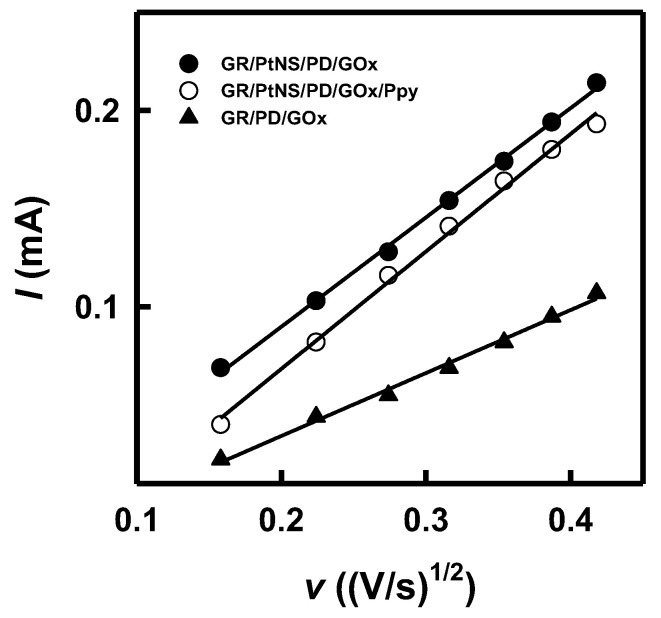
The relationships between square root of scan rate and the registered peak anodic current for GR/PD/GOx, GR/PtNS/PD/GOx, and GR/PtNS/PD/GOx/Ppy electrodes.

The EASA of GR/PD/GOx, GR/PtNS/PD/GOx, and GR/PtNS/PD/GOx/Ppy electrodes was calculated using the relationship between the square root of the scan rate and the registered peak anodic current (Figure 3) and the Randles–Sevcik equation (Equation (2)). The Ru(NH_3_)_6_Cl_3_ was used as a redox probe because its oxidation occurs in the diapason, where developed electrodes do not give an additional signal (Figure S2). In the case of GR/PD/GOx, GR/PtNS/PD/GOx, and GR/PtNS/PD/GOx/Ppy electrodes, EASA was 0.397, 0.689, and 0.741 cm^2^, respectively. Modification of GR/PD/GOx electrodes with PtNS increased the EASA more than 1.74 times.

### 3.2. The Investigation of Glucose Biosensors Based on Differently Modified Electrodes

**Figure 4:**
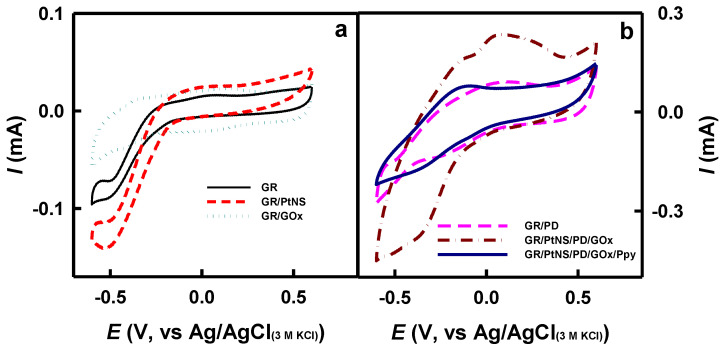
The cyclic voltammograms of glucose biosensors without (**a**) and with (**b**) redox mediator—PD. Cyclic voltammograms were recorded in 0.05 M SA buffer (pH 6.0) with 0.1 M KCl.

The electroactivity of the modified electrodes in the absence (Figure 4a) and in the presence (Figure 4b) of redox mediator PD was investigated using the CV method as described in Section 2.4. In the case of bare GR, GR/PtNS, and GR/GOx electrodes (Figure 4a), smooth, peak-free cyclic voltammograms were recorded. However, distinct anodic and cathodic peaks appeared in the presence of a redox mediator for GR/PD, GR/PtNS/PD/GOx, and GR/PtNS/PD/GOx/Ppy (Figure 4b) electrodes.

Cyclic voltammograms registered using GR/PtNS/PD/GOx and GR/PtNS/PD/GOx/Ppy electrodes were characterized by anodic and cathodic peaks. Anodic peaks of GR/PtNS/PD/GOx and GR/PtNS/PD/GOx/Ppy electrodes were noticeable at +0.048 and −0.16 V vs. Ag/AgCl_(3M KCl)_, respectively. The future investigations of glucose biosensors based on developed electrodes were performed using the potential of +0.30 V vs. Ag/AgCl_(3M KCl)_.

**Figure 5:**
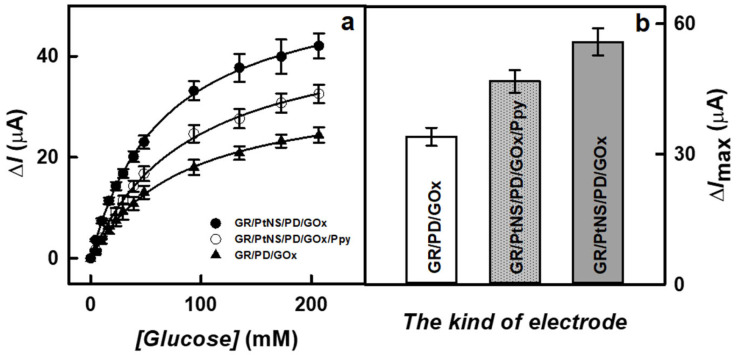
The calibration plots (**a**) and current responses recorded at 207 mM glucose (**b**) of glucose biosensors based on GR/PtNS/PD/GOx, GR/PtNS/PD/GOx/Ppy and GR/PD/GOx electrodes. Measurements were performed at +0.30 V vs. Ag/AgCl_(3M KCl)_ in 0.05 M SA buffer (pH 6.0) with 0.1 M KCl; the number of measurements (*n*) is 3.

The presence of nanomaterials and the formation of a polymer layer on the surface of the working electrode play a significant role in the sensitivity of biosensors. To evaluate the effect of PtNS and Ppy on the current response to glucose, studies were performed using GR/PtNS/PD/GOx and GR/PtNS/PD/GOx/Ppy electrodes in 0.05 M SA buffer (pH 6.0) with 0.1 M KCl by increasing the concentration of glucose up to 207 mM using CPA method. The results obtained were compared with those recorded using the GR/PD/GOx electrode. Hyperbolic dependences of current responses on the concentration of glucose are presented in Figure 5a.

As can be seen in Figure 5b, the presence of PtNS allowed for an increase in the registered current responses. The Δ*I*_max_ (55.8 ± 3.1 μA) in the case of the GR/PtNS/PD/GOx electrode was 1.64 times higher than for the GR/PD/GOx electrode (34.0 ± 2.1 μA). This difference can be explained by the larger electroactive surface area in the case of PtNS. The Δ*I*_max_ calculated for GR/PtNS/PD/GOx/Ppy was 46.8 ± 2.6 μA. The reduction of Δ*I*_max_ could be attributed to the hindered electron transfer through the Ppy layer to the surface of the working electrode. Moreover, the glucose biosensor based on an electrode modified with a polymer layer was characterized by a high *K*_M(app)_, which is an important parameter for glucose analysis in real samples. The values of *K*_M(app)_ for GR/PtNS/PD/GOx and GR/PtNS/PD/GOx/Ppy electrodes were 67.1 and 89.8 mM, respectively.

### 3.3. The Evaluation of Analytical Characteristics of Glucose Biosensors

**Figure 6:**
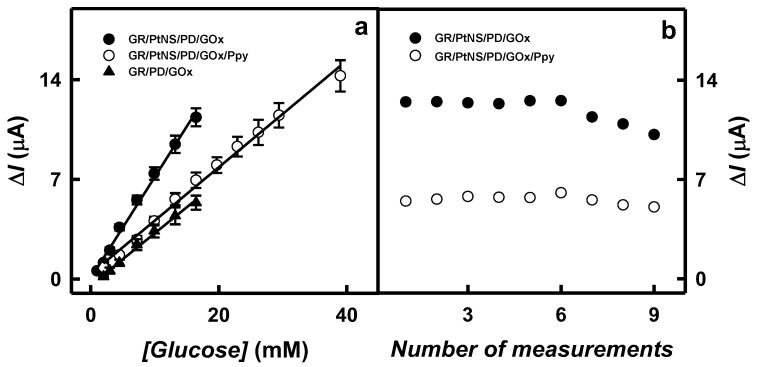
The linear range for GR/PtNS/PD/GOx, GR/PtNS/PD/GOx/Ppy, and GR/PD/GOx electrodes (**a**) and the reproducibility of GR/PtNS/PD/GOx, GR/PtNS/PD/GOx/Ppy electrodes (**b**). Reproducibility was tested by measuring 9.92 mM of glucose.

The LR characterized for glucose biosensors based on GR/PtNS/PD/GOx and GR/PtNS/PD/GOx/Ppy electrodes are presented in Figure 6a. To establish the advantage of electrodes modified with PtNS and Ppy, the achieved results were compared with those obtained with the GR/PD/GOx electrode. As shown in Figure 6a, the application of Ppy allowed extending the LR up to 39.0 mM, which was 2.36 times wider than that for GR/PtNS/PD/GOx and GR/PD/GOx electrodes (up to 16.5 mM).

The glucose biosensors based on GR/PtNS/PD/GOx and GR/PtNS/PD/GOx/Ppy electrodes were characterized by good reproducibility of 5.93 and 6.19% (4.48 mM of glucose) and good repeatability of 7.38 and 5.50% for nine measurements (Figure 6b). The 95% current responses with GR/PtNS/PD/GOx and GR/PtNS/PD/GOx/Ppy electrodes (5 s) were recorded 2.2 times faster than with the GC/OOPpy_(300s)_-GNPs/GOx electrode (11 s) [13].

The properties of developed biosensors were compared to other glucose biosensors based on nanomaterials (Table 1). The investigated LR of glucose biosensor based on GR/PtNS/PD/GOx electrode was 5.5 and 1.65 times wider in comparison to LR obtained using an enzymatic biosensor based on LSG/HEC-PtNPs/GOx (up to 3 mM) [24] and GR/GNPs_(3.5nm)_/PD/GOx (up to 10 mM) [16], respectively.

The evaluated LR of the GR/PtNS/PD/GOx/Ppy electrode was 4.88 and 4.81 times wider than for glassy carbon (GC) electrode with electrodeposited overoxidized polypyrrole (OOPpy_(300s)_) film decorated with GNPs and immobilized GOx (GC/OOPpy_(300s)_-GNPs/GOx, up to 8.0 mM) [13] and for GC electrode decorated with hybrid nanomaterial of polyamidoamine dendrimer (PAMAM), (3-glycidyloxypropyl)trimethoxysilane (Sil), reduced graphene oxide (rGO), and deposited PtNPs_(3.3nm)_ and GOx (GC/PAMAM-Sil-rGO/PtNPs_(3.3nm)_/GOx, up to 8.1 mM) [21], respectively. The LR obtained for the glucose biosensor based on the Pt electrode modified with polyvinylferrocenium perchlorate (PVF^+^ClO_4_^−^), PtNPs_(25nm)_ and immobilized poly-*o*-phenylenediamine (p*o*PD) and GOx (Pt/PVF^+^ClO_4_^−^/PtNPs_(25nm)_/p*o*PD-GOx, up to 9.64 mM) was 4.05 times narrow than for the GR/PtNS/PD/GOx/Ppy electrode presented here [14]. The developed glucose biosensor based on the GR/PtNS/PD/GOx/Ppy electrode was characterized by 2.17 and 27.9 times wider values of LR than glucose biosensors based on ITO/PtNCs_(1.76nm)_/UiO-66-*g*-P2VP electrode (up to 18 mM) [33] and based on GC electrode covered with a hybrid of multi-walled carbon nanotubes (MWCNT) and lignosulfonate (LS) decorated with PtNPs and modified with polyethyleneimine (PEI) and GOx (GC/MWCNT/LS/PtNPs_(11.07nm)_/PEI/GOx (up to 1.4 mM) [31].

Presented in this study glucose biosensor based on the GR/PtNS/PD/GOx electrode was 1.90 times more sensitive (10.1 μA/(mM cm^2^)) than that based on the GR/PtNS/PD/GOx/Ppy electrode (5.31 μA/(mM cm^2^)). The sensitivity of GR/PtNS/PD/GOx/Ppy electrode was 1.11 times higher in comparison with the results obtained using the GC/MWCNT/LS/PtNPs/PEI/GOx electrode (4.77 μA/(mM cm^2^)) [31] and 1.75 times higher compared to the GR/DGNs/(PD/GOx)_3_/Ppy electrode (3.03 μA/(mM cm^2^) [43]. The values of LOD for glucose biosensors based on GR/PtNS/PD/GOx and GR/PtNS/PD/GOx/Ppy electrodes were evaluated as 0.198 and 0.561 mM, respectively. While the Ppy layer on the surfaces of modified working electrodes reduces the sensitivity of the developed biosensor, it can still be effectively employed for the biosensing of glucose in real samples due to their wide linear range.

### 3.4. The Storage Stability and the Application of Developed Biosensors for Glucose Determination in Blood Serum

**Figure 7:**
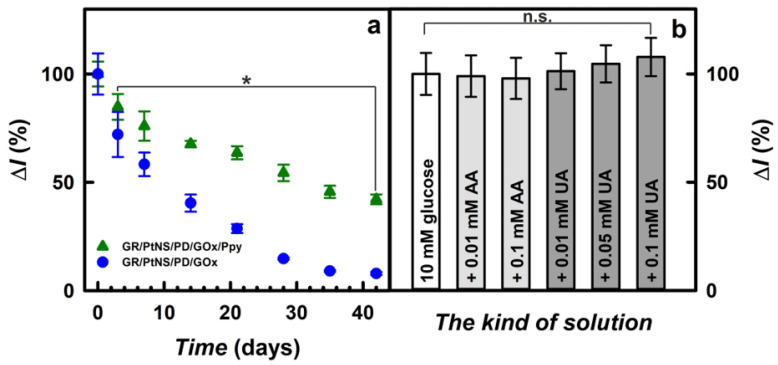
Time variation of current responses (207 mM of glucose) for glucose biosensors based on GR/PtNS/PD/GOx or GR/PtNS/PD/GOx/Ppy electrodes (**a**). The effect of electroactive species in the serum sample on the current responses using glucose biosensor based on GR/PtNS/PD/GOx/Ppy electrode (**b**). Current responses were registered in 0.05 M SA buffer (pH 6.0) with 0.1 M KCl (**a**) and in a 10-times diluted sample of blood serum (**b**) at +0.30 V vs. Ag/AgCl_(3M KCl)_, *n* not less as 3. Asterisk denotes significant differences compared to day 0 (*p* < 0.05). n.s.—non-significant differences (*p* < 0.05).

The subsequent step of this study was the investigation of the storage stability of fabricated glucose biosensors. Electrodes between measurements were stored at +4 °C over the SA buffer solution (pH 6.0) for up to 42 days. As is seen in Figure 7a, the values of current responses for the glucose biosensor based on the GR/PtNS/PD/GOx/Ppy electrode were decreased gradually; this could be explained by the inactivation of the enzyme and the degradation of polymers [10]. Meanwhile, the decrease in the current responses registered using the GR/PtNS/PD/GOx electrode was sharper than for the GR/PtNS/PD/GOx/Ppy electrode.

Glucose biosensors based on GR/PtNS/PD/GOx and GR/PtNS/PD/GOx/Ppy electrodes retained 50% of their initial current responses (*t*_1/2_) after 9 and 31 days, respectively. It is evident that the GR/PtNS/PD/GOx/Ppy electrode is 3.44 times more stable than the GR/PtNS/PD/GOx electrode and could be employed for glucose biosensing. The developed glucose biosensor based on the GR/PtNS/PD/GOx/Ppy electrode was 2.21 times more stable than the biosensors based on the GC/PAMAM-Sil-rGO/PtNPs_(3.3nm)_/GOx (*t*_1/2_ = 14 days) [21] electrode. The GR/PtNS/PD/GOx and GR/PtNS/PD/GOx/Ppy electrodes were more stable compared to the GR/DGNs/(PD/GOx)_3_ (*t*_1/2_ = 2.0 days) and GR/DGNs/(PD/GOx)_3_/Ppy (*t*_1/2_ = 9.0 days) electrodes [43]. The remarkable storage stability was considered to be the advantage of the GR/PtNS/PD/GOx/Ppy electrode, where PtNS combined with Ppy created a favorable microenvironment for GOx immobilization.

The presence of a polymer layer serves as an effective permselective barrier to circumvent interfering compounds in blood samples [16]. The selectivity of glucose biosensors based on the GR/PtNS/PD/GOx/Ppy electrode toward interfering (sugars) and electroactive (ascorbic and uric acids) species that caused errors in glucose measurements [5,50] was investigated in a 10-times diluted sample of blood serum using the procedures outlined in Section 2.5. The developed glucose biosensor based on the GR/PtNS/PD/GOx/Ppy electrode showed good discrimination against 1.00 mM of sugars (Figure S3), indicating the suitability for glucose detection.

Uric acid in the presence of ascorbic acid in the plasma provides antioxidation protection for the cardiovascular system’s cells [51]. Having antioxidant properties in the hydrophilic environment, ascorbic and uric acids [50,51,52] are oxidized in biological samples at a relatively positive potential and are interfering with the detection of glucose [40]. Normal physiological levels of AA and UA in human blood serum do not exceed 0.141 mM [53] and 0.1 mM [30], respectively. As shown in Figure 7b, the current response after the addition of 10.0 mM glucose with 0.01 or 0.1 mM of AA decreased for the GR/PtNS/PD/GOx/Ppy electrode by 1.0 and 2.1% compared to results without AA. The impact (6.4%) of 0.001 mM AA registered in the case of the GC/PAMAM-Sil-rGO/PtNPs_(3.3nm)_/GOx electrode was higher compared to the developed biosensor [21].

Meanwhile, the effect of uric acids on the performance of the developed glucose biosensors was more significant. The current responses registered using the GR/PtNS/PD/GOx/Ppy electrode after the addition of 10.0 mM glucose with 0.01, 0.05, or 0.1 mM of UA increased by 1.0, 5.0, or 8.0%, respectively, compared to the results in the absence of UA. It is evident that the glucose biosensor based on the GR/PtNS/PD/GOx/Ppy electrode developed in this study is quite resistant to electroactive species. However, it should be noted that the registered signals after the addition of AA or UA were not significantly different from those observed without these electroactive species.

Normally, glucose concentration in the blood of non-diabetic individuals does not exceed 5.5 mM [13] and does not exceed 30 mM in diabetics [2]. The GR/PtNS/PD/GOx/Ppy electrode was used to determine glucose in real blood serum samples. Measurements were performed in a 10-times diluted blood serum with 0.708 mM of glucose with the addition method. The glucose concentration in a 10-times diluted blood serum was evaluated as 0.676 ± 0.032 mM with 95.5% of the recovery ratio (Figure S4). The developed biosensor based on the GR/PtNS/PD/GOx/Ppy electrode was applied for glucose analysis in a 10-times diluted samples of human serum, and the achieved results are supplied in Table 2.

It was evaluated that the recovery ratio of glucose biosensors based on the GR/PtNS/PD/GOx/Ppy electrode was in the range from 95.7 ± 7.9% to 96.9 ± 6.0% and is at the same level as the commercial glucose sensors. The allowed recovery ratio and relative error can vary depending on the amount of glucose, the method of determination, the country, and the agency involved. The determination criterion for ≥75 mg/dL (4.17 mM) of glucose in the USA is 98 ± 15%, and for ≥100 mg/dL (5.55 mM) of glucose in Europe—95 ± 15% [54].

It can be argued that the developed glucose biosensors based on GR/PtNS/PD/GOx and GR/PtNS/PD/GOx/Ppy electrodes have the following advantages: (i) wide linear glucose determination range (up to 16.5 and 39.0 mM) and good reproducibility (5.93 and 6.19%); (ii) high storage stability and short duration of measurements (less than 5 s); (iii) sufficient sensitivity (10.1 and 5.31 μA/(mM cm^2^)) and limit of detection (0.198 and 0.561 mM); (iv) high resistance to electroactive species and suitability for glucose analysis (95.7–96.9% for GR/PtNS/PD/GOx/Ppy) in the samples of blood serum. In clinical practice, the application of the GR/PtNS/PD/GOx/Ppy electrode might be more successful when compared to the GR/PtNS/PD/GOx electrode owing to its sensitivity, stability, and anti-interfering capability.

## 4. Conclusions

In this paper, a reagentless electrochemical glucose biosensor was developed immobilizing the redox mediator on the electrode and combining the advantages of platinum nanostructures and polypyrrole layers. The PtNS increase the surface of the working electrode more than 1.74 times, which enhances the electron transfer efficiency and the sensitivity of the glucose biosensor. The glucose biosensor was characterized by simple operation, cost-effectiveness, rapid response, wide linear range (up to 39.0 mM), good stability (*t*_1/2_ = 31 days), and well performance in the presence of interfering compounds in the blood serum. All these advantages allow us to conclude that the developed biosensor well meets the requirements of an analytical system used for the control of glucose concentration in the blood of people with diabetes. Incidentally, a methodology presented here demonstrates the opportunities and benefits of creating novel hybrid devices relevant to a variety of applications, including clinical and environmental assays as well as general diagnostic purposes.

## Figures and Tables

**Table 1:** The comparison of analytical characteristics for glucose biosensors.

Working Electrode	LOD (mM)/Sensitivity (μA/(mM cm^2^))	LR (mM)	Reference
LSG/HEC-PtNPs/GOx	0.00023/69.64	0.005–3	[24]
CG/CNT/PtNPs_(2–3nm)_/GOx	0.0005/–	0.0005–5	[27]
ITO/PtNCs_(1.76nm)_/UiO-66-*g*-P2VP	0.00218/199.58 0.00218/74.45	0.01–5 5–18	[33]
GC/MWCNT/LS/PtNPs_(11.07nm)_/PEI/GOx	0.01567/4.77	0.050–1.4	[31]
Pt/PVF^+^ClO_4_^−^/PtNPs_(25nm)_/p*o*PD-GOx	0.018/17.40	0.06–9.64	[14]
GR/GNPs_(3.5nm)_/PD/GOx	0.024/52.1	0.1–10.0	[16]
GC/OOPpy_(300s)_-GNPs/GOx	0.5/–	1.0–8.0	[13]
GR/DGNs/(PD/GOx)_3_/Ppy	0.683/3.03	2.0–39.0	[43]
GC/PAMAM-Sil-rGO/PtNPs_(3.3nm)_/GOx	0.8/24.6	0.01–8.1	[21]
GR/PtNS/PD/GOx	0.198/10.1	1.00–16.5	This work
GR/PtNS/PD/GOx/Ppy	0.561/5.31	2.00–39.0	This work

DGNs—dendritic gold nanostructures, GC—glassy carbon, HEC—hydroxyethyl cellulose, ITO—indium tin oxide, LS—lignosulfonate, LSG—laser-scribed graphene, MWCNT—multi-walled carbon nanotubes, OOPpy_(300s)_—overoxidized polypyrrole, PAMAM—polyamidoamine dendrimer, PEI—polyethyleneimine, p*o*PD—poly-*o*-phenylenediamine, PtNCs—platinum nanoclusters, P2VP—poly(2-vinylpyridine), PVF^+^ClO_4_^−^—polyvinylferrocenium perchlorate, rGO—reduced graphene oxide, Sil—(3-glycidyloxypropyl)trimethoxysilane, UiO-66-*g*—grafted metal organic framework.

**Table 2:** The main results of measurements in a 10-times diluted samples of blood serum with 0.708 mM of glucose using biosensor based on GR/PtNS/PD/GOx/Ppy electrode.

Concentration of Glucose (mM)		Recovery Ratio (%)
Total	Detected * (*n* = 3)	
2.81	2.69 ± 0.20	95.7
7.11	6.85 ± 0.51	96.3
14.8	14.3 ± 0.9	96.6
19.2	18.6 ± 1.1	96.9

* Responses of CPA were registered in a 10-times diluted serum at +0.30 V vs. Ag/AgCl_(3M KCl)_.

## Data Availability

Data are contained within the article.

## Supplementary Materials

The following supporting information can be downloaded at: https://www.mdpi.com/article/10.3390/bios14030134/s1, Figure S1: Cyclic voltammogram registered using GR/PtNS electrode in 0.5 M H_2_SO_4_ solution; Figure S2: Cyclic voltammograms of GR/PD/GOx (a), GR/PtNS/PD/GOx (b), and GR/PtNS/PD/GOx/Ppy (c) electrodes recorded at scan rates from 0.025 to 0.175 V/s in the solution of 1 mM Ru(NH_3_)_6_Cl_3_ with 0.1 M KCl; Figure S3: The impact of sugars on the current response of glucose biosensors based on the GR/PtNS/PD/GOx/Ppy electrode. An amperogram was registered in a 10-times diluted sample of blood serum after the addition of 10.0 mM glucose and 1.0 mM of various sugars at +0.30 V vs. Ag/AgCl_(3M KCl)_; Figure S4: The determination of glucose in blood serum. Measurements were performed at +0.30 V vs. Ag/AgCl_(3M KCl)_ in a 10-times diluted sample of blood serum with 0.708 mM of glucose using the addition method.
