# Supplementary material for: Reagentless Glucose Biosensor Based on Combination of Platinum Nanostructures and Polypyrrole Layer
Source: Biosensors (Basel). 2024 Mar 4;14(3):134. doi: 10.3390/bios14030134 (PMC10968463; doi:10.3390/bios14030134)
Supplement: Supplementary file 1 [file biosensors-14-00134-s001.zip › biosensors-2864441-supplementary.pdf]

Reagentless Glucose Biosensor Based on Combination of Platinum Nanostructures and Polypyrrole Layer

Natalija German [1,*], Anton Popov [1,2] and Almira Ramanaviciene [1,2,*]

[1] Department of Immunology and Bioelectrochemistry, State Research Institute Centre for Innovative Medicine, Santariskiu 5, LT-08406, Vilnius, Lithuania

[2] NanoTechnas—Center of Nanotechnology and Materials Science, Institute of Chemistry, Faculty of Chemistry and Geosciences, Vilnius University, Naugarduko str. 243, LT-03225 Vilnius, Lithuania

* Correspondence: natalija.german@imcentras.lt (N.G.); almira.ramanaviciene@chf.vu.lt (A.R.)

Supplementary data

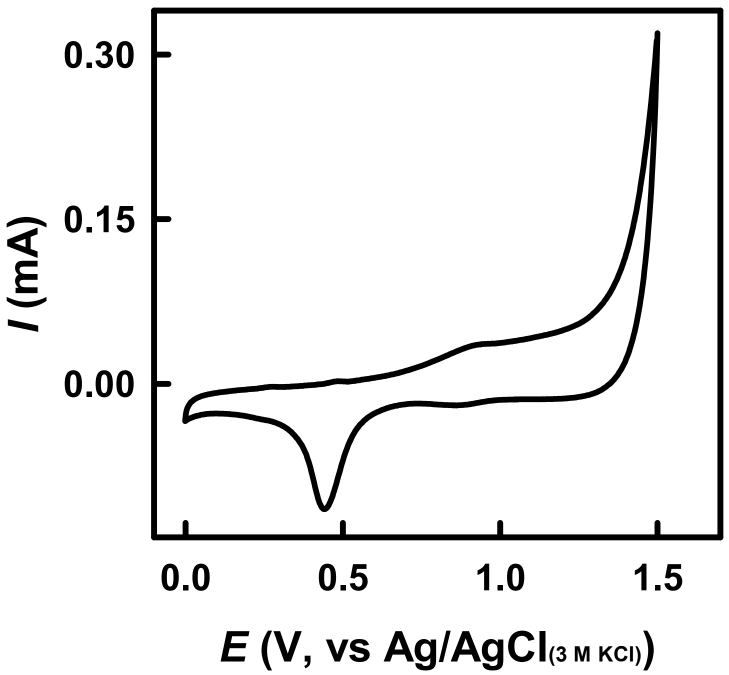

Figure S1. Cyclic voltammogram registered using GR/PtNS electrode in 0.5 M H_2SO_4 solution.

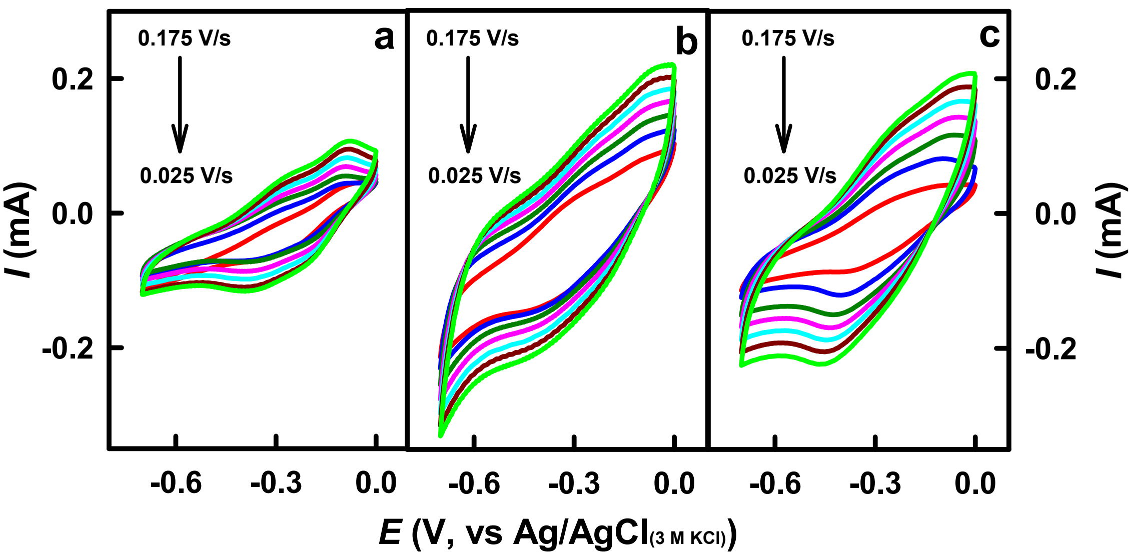

Figure S2. Cyclic voltammograms of GR/PD/GOx (**a**), GR/PtNS/PD/GOx (**b**), and GR/PtNS/PD/GOx/Ppy (**c**) electrodes recorded at scan rates from 0.025 to 0.175 V/s in the solution of 1 mM $Ru(NH_3)_6Cl_3$ with 0.1 M KCl.

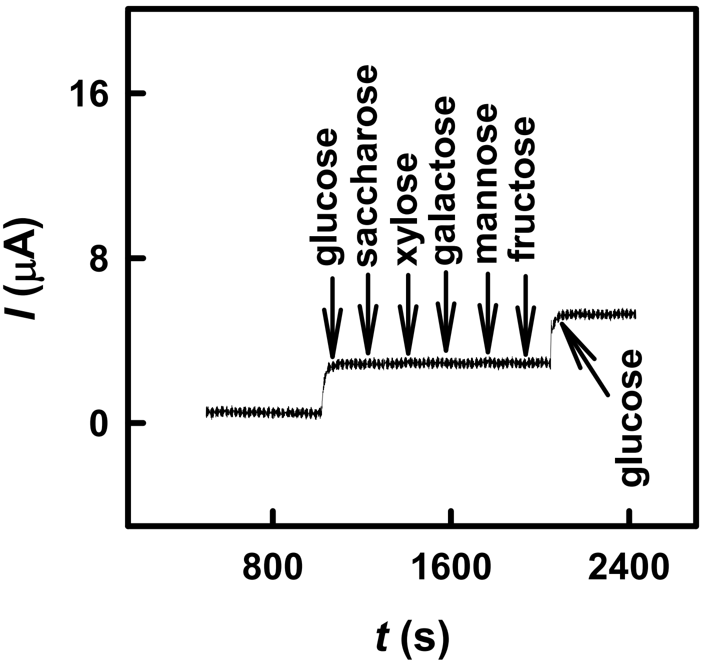

Figure S3. The impact of sugars on the current response of glucose biosensors based on the GR/PtNS/PD/GOx/Ppy electrode. An amperogram was registered in a 10-times diluted sample of blood serum after the addition of 10.0 mM glucose and 1.0 mM of various sugars at +0.30 V vs. Ag/AgCl(3M KCl).

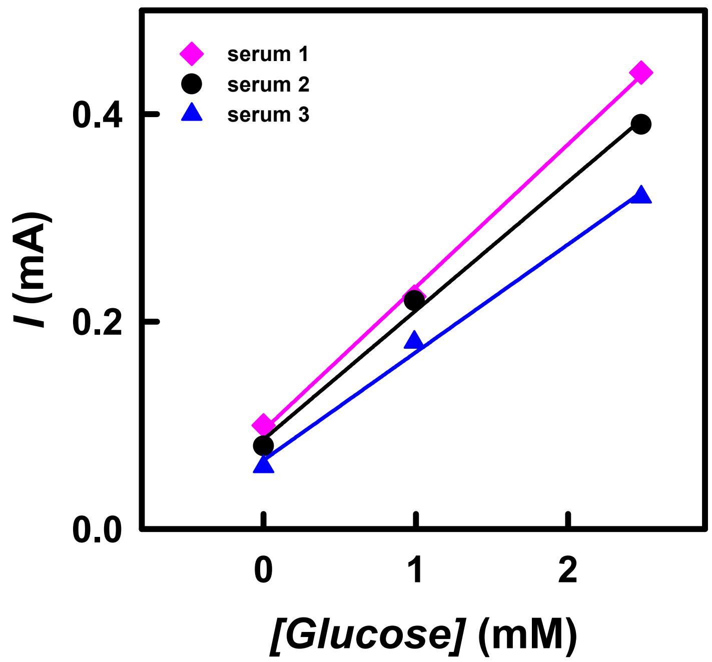

Figure S4. The determination of glucose in blood serum. Measurements were performed at +0.30 V vs. Ag/AgCl$_{(3M\ KCl)}$ in a 10-times diluted sample of blood serum with 0.708 mM of glucose using the addition method.
